# COVID-19-Associated Cardiovascular Complications: A Narrative Literature Review

**DOI:** 10.7759/cureus.105394

**Published:** 2026-03-17

**Authors:** Aashna Lala, Mykhailo Vysochyn, Kateryna Shyshkova

**Affiliations:** 1 Clinical Sciences, Saint James School of Medicine, Park Ridge, USA; 2 Basic Sciences, Saint James School of Medicine, Arnos Vale, VCT

**Keywords:** cardiac arrhythmias, cardiovascular complications, covid-19, heart failure, hypercoagulability, hypertension, myocardial injury, myocarditis, sars-cov-2, thromboembolism

## Abstract

Since the emergence of severe acute respiratory syndrome coronavirus 2 (SARS-CoV-2) in 2019, coronavirus disease 2019 (COVID-19) has caused significant global morbidity and mortality. Although initially recognized as a respiratory illness, COVID-19 is now understood to be a systemic disease with substantial cardiovascular involvement. Both patients with pre-existing cardiovascular disease and those without prior cardiac conditions may develop a wide range of cardiovascular complications during and after acute infection.

Reported complications include myocardial injury, myocarditis, heart failure, arrhythmias, and thromboembolic events, all of which contribute to increased disease severity and mortality. This narrative review summarizes current evidence on cardiovascular complications associated with COVID-19 among adult patients in the United States, with particular attention to differences between individuals with pre-existing cardiovascular disease and those who develop new cardiovascular pathology following SARS-CoV-2 infection. This review discusses proposed mechanisms of cardiovascular injury, clinical manifestations, diagnostic approaches, and current management strategies. By summarizing current knowledge, this review aimed to increase awareness of COVID-19-related cardiovascular complications, support timely recognition in emergency and inpatient settings, and assist clinicians in improving patient outcomes.

## Introduction and background

The COVID-19 pandemic has profoundly transformed contemporary medicine and highlighted the multisystem effects of SARS-CoV-2 infection [1]. Although early clinical descriptions emphasized respiratory manifestations, such as pneumonia and acute respiratory distress syndrome, it quickly became evident that COVID-19 significantly affects the cardiovascular system. Cardiac complications emerged as major contributors to morbidity and mortality, particularly among hospitalized and critically ill patients [2,3].

Individuals with pre-existing chronic conditions, including hypertension, coronary artery disease, heart failure, diabetes mellitus, and obesity, consistently demonstrated worse outcomes following SARS-CoV-2 infection [4,5]. These comorbidities increased vulnerability to severe disease and heightened the risk of acute cardiovascular decompensation during infection [4,5]. However, significant cardiac complications were also observed in patients without prior cardiovascular disease. Many developed acute myocardial injury, myocarditis, arrhythmias, or heart failure as a direct or indirect consequence of infection, underscoring the cardiotoxic potential of SARS-CoV-2 [6,7].

Several mechanisms have been proposed to explain cardiovascular involvement in COVID-19. SARS-CoV-2 enters host cells through the angiotensin-converting enzyme 2 (ACE2) receptor, which is expressed in cardiac myocytes and vascular endothelial cells [5,6]. Viral entry, combined with exaggerated immune activation and systemic inflammation, contributes to myocardial injury and endothelial dysfunction [5-7]. Cytokine-mediated inflammation promotes thrombosis and microvascular injury, increasing the risk of ischemia and thromboembolic complications [8-10]. These mechanisms may overwhelm cardiac compensatory capacity, particularly in individuals with limited cardiovascular reserve.

Heart failure has emerged as a growing concern during the pandemic. Patients with established heart failure frequently experience acute decompensation during infection due to hypoxia, metabolic stress, inflammatory myocardial depression, and fluid shifts [11]. Additionally, new-onset heart failure has been reported in previously healthy individuals with severe COVID-19 [11,12]. Thromboembolic complications further contribute to cardiovascular instability and adverse outcomes [9,10].

Despite growing recognition of cardiovascular involvement, important gaps remain in understanding the extent and mechanisms of cardiac injury in COVID-19. This narrative review, therefore, examines the spectrum of cardiovascular complications associated with acute SARS-CoV-2 infection, focusing on both pre-existing disease and new-onset cardiac pathology.

This narrative review is based on peer-reviewed publications indexed in PubMed between 2020 and 2022, with a focus on adult populations and acute cardiovascular complications associated with SARS-CoV-2 infection. Emphasis was placed on clinically relevant studies, major society statements, and high-impact observational data to provide an integrated overview of current evidence.

## Review

Pathophysiology of COVID-19-associated cardiovascular injury

Viral Entry and Direct Myocardial Injury

The cardiovascular impact of COVID-19 results from a complex interaction between direct viral myocardial injury, immune dysregulation, endothelial dysfunction, hypercoagulability, and systemic hemodynamic stress [5-7,9,10].

SARS-CoV-2 gains entry into host cells by binding to the angiotensin-converting enzyme 2 (ACE2) receptor, a key regulator of the renin-angiotensin-aldosterone system. ACE2 is expressed not only in pulmonary tissue but also in cardiomyocytes, cardiac fibroblasts, and vascular endothelial cells [5,6]. Viral entry leads to downregulation of ACE2, disrupting the balance between vasodilatory and vasoconstrictive pathways and promoting inflammation, oxidative stress, and fibrosis mediated by angiotensin II [6,7]. Histopathological and autopsy studies have demonstrated endothelial injury, microvascular thrombosis, and cardiac pathology in severe disease [11,13,14]. Clinically, this may present as myocarditis, heart failure, arrhythmias, or cardiogenic shock [6,7]. However, myocardial injury has also been observed without clear histologic evidence of classic myocarditis, suggesting additional indirect mechanisms of injury [15].

Immune Dysregulation and Cytokine-Mediated Injury

Severe COVID-19 is frequently associated with dysregulated immune activation and excessive cytokine release. Elevated proinflammatory mediators contribute to systemic inflammation and myocardial depression [6,7]. Cytokine-mediated injury impairs myocardial contractility and may precipitate ventricular dysfunction, particularly in patients with limited cardiac reserve [6,11]. Inflammation also increases myocardial oxygen demand while hypoxia and microvascular dysfunction reduce oxygen delivery, further exacerbating myocardial stress [6,7,9,10]. Importantly, myocardial injury may occur without demonstrable direct viral cytotoxicity in cardiomyocytes, highlighting the central role of inflammation and systemic illness severity [6,7].

Endothelial Dysfunction and Microvascular Injury

Endothelial dysfunction is a critical contributor to COVID-19-associated cardiovascular complications. Endothelial injury and loss of antithrombotic function have been described in severe disease, contributing to microvascular dysfunction and organ injury [14]. Impaired nitric oxide bioavailability reduces vasodilation and increases susceptibility to ischemia [6,7]. Endothelial injury within coronary vessels may result in myocardial ischemia without obstructive coronary disease, while pulmonary vascular involvement can increase pulmonary vascular resistance and contribute to right ventricular strain and failure [6,14].

Hypercoagulability and Thromboembolic Risk

COVID-19 is associated with a marked hypercoagulable state characterized by elevated D-dimer levels, fibrinogen abnormalities, platelet activation, and impaired fibrinolysis [9,10]. Endothelial injury and systemic inflammation amplify thrombotic risk [9,10,14,15]. Pulmonary embolism is among the most frequently reported thrombotic complications and may result in acute right ventricular failure and rapid clinical deterioration [16-18]. Arterial thromboses, including acute coronary syndromes and ischemic stroke, have also been described, reflecting both plaque destabilization and in situ thrombosis in a prothrombotic milieu [6,9,10]. Microvascular thrombosis further contributes to myocardial injury by impairing perfusion at the microcirculatory level [14].

Hemodynamic Stress and Demand Ischemia

Severe SARS-CoV-2 infection imposes substantial metabolic and hemodynamic stress. Fever, tachycardia, systemic inflammation, and hypoxemia increase myocardial oxygen demand while limiting supply. This imbalance may lead to type 2 myocardial infarction, particularly in patients with pre-existing coronary artery disease [19]. Intravascular volume shifts, renal dysfunction, and positive pressure ventilation further strain cardiovascular function [4,6]. Patients with heart failure, valvular disease, or pulmonary hypertension are especially vulnerable to rapid clinical deterioration under these conditions [4,11].

Implications for Heart Failure Development

The combined effects of inflammatory myocardial depression, endothelial dysfunction, thrombosis, and hemodynamic stress create a setting conducive to both exacerbation of existing heart failure and development of new cardiomyopathy [6,7,11,12]. Individuals without prior cardiovascular disease may develop transient or persistent ventricular dysfunction following infection, whereas those with established heart failure are at high risk for rapid decompensation [11,12].

Cardiovascular Complications in Patients With Pre-existing Cardiovascular Disease

Patients with established cardiovascular disease represent a particularly vulnerable population during acute SARS-CoV-2 infection. Comorbid conditions such as hypertension, coronary artery disease, heart failure, diabetes mellitus, and obesity have consistently been associated with increased disease severity, higher hospitalization rates, and worse clinical outcomes [4-6]. Systemic inflammation and hemodynamic stress frequently precipitate acute cardiovascular decompensation in these individuals [4,6,11].

Hypertension and Vascular Disease

Hypertension is among the most common comorbidities observed in hospitalized COVID-19 patients. Although hypertension does not necessarily increase susceptibility to SARS-CoV-2 infection, it is associated with severe disease and adverse outcomes in hospitalized populations [4-6]. SARS-CoV-2-mediated perturbation of ACE2 pathways may further disrupt vascular homeostasis and contribute to endothelial injury [6,8].

Coronary Artery Disease and Acute Coronary Syndromes

Patients with pre-existing coronary artery disease are susceptible to myocardial injury during COVID-19 infection. Systemic inflammation increases myocardial oxygen demand, while hypoxemia and microvascular dysfunction reduce oxygen supply, creating a supply-demand mismatch [6,19]. Inflammation may also destabilize atherosclerotic plaques, predisposing to acute coronary syndromes [20,21]. Elevated troponin levels in this population are strongly associated with increased mortality [3,22].

Heart Failure Exacerbation

Heart failure is one of the most significant cardiovascular comorbidities influencing COVID-19 outcomes. Both heart failure with reduced ejection fraction and preserved ejection fraction are associated with increased risk of decompensation during acute infection [11,12]. Contributors include hypoxia, systemic inflammation, increased metabolic demand, sodium and water retention, and neurohormonal activation [11,12]. Patients with heart failure are more likely to require intensive care and have higher mortality rates [11,12].

Diabetes Mellitus and Obesity

Diabetes mellitus and obesity frequently coexist with cardiovascular disease and independently increase the risk of severe COVID-19 infection [4-6]. Chronic inflammation, insulin resistance, endothelial dysfunction, and impaired immune responses contribute to increased disease severity [5,6].

Arrhythmias in Patients With Underlying Cardiac Disease

Patients with structural heart disease are at increased risk for atrial and ventricular arrhythmias during COVID-19 infection. Myocardial inflammation, ischemia, hypoxia, and electrolyte disturbances contribute to arrhythmogenesis. Atrial fibrillation is the most frequently reported arrhythmia, and ventricular arrhythmias, though less common, are associated with poor prognosis [23].

Clinical Outcomes and Prognostic Implications

Multiple cohort studies demonstrate that underlying cardiovascular disease and myocardial injury are independently associated with mortality among hospitalized COVID-19 patients [3,4,6,22]. These findings underscore the need for early identification and close monitoring of patients with pre-existing cardiovascular disease during acute SARS-CoV-2 infection [4,6].

New-onset cardiovascular disease following COVID-19 infection

Acute Myocardial Injury

Acute myocardial injury, defined by elevated cardiac troponin levels, is common in hospitalized COVID-19 patients [3,12,22]. Meta-analytic data support the prognostic significance of troponin elevation in COVID-19 [24]. Mechanisms include cytokine-mediated injury, microvascular ischemia, and demand ischemia [6,7,19]. Even mild troponin elevations correlate with worse outcomes [3,12,22].

Myocarditis

Myocarditis is a recognized but relatively uncommon manifestation of COVID-19 [15,25]. Cardiac magnetic resonance (CMR) findings, such as edema and late gadolinium enhancement, can support diagnosis and characterize myocardial injury patterns [26-29]. Diagnostic frameworks emphasize clinical context, biomarkers, imaging, and exclusion of alternate etiologies [30-33]. Clinical recovery varies, with some patients demonstrating persistent myocardial changes on follow-up imaging [26,27,34].

New-Onset Heart Failure and Cardiomyopathy

New-onset heart failure has been described in hospitalized COVID-19 patients, particularly among critically ill individuals [11,12]. Mechanisms include inflammatory myocardial depression, myocarditis-related dysfunction, stress-induced cardiomyopathy, and unmasking of subclinical disease [6,7,11,12]. Long-term implications of new-onset cardiomyopathy remain under investigation, including post-acute cardiovascular risk signals.

Cardiac Arrhythmias

Arrhythmias are observed in patients without prior cardiac disease, most commonly atrial fibrillation [23]. Persistent arrhythmias beyond acute infection may necessitate long-term follow-up [23].

Thromboembolic Events in Previously Healthy Individuals

COVID-19-associated hypercoagulability may lead to venous and arterial thrombosis even in patients without pre-existing cardiovascular disease [9,10,16-18]. These findings support maintaining a high index of suspicion for thromboembolic complications in patients presenting with unexplained cardiopulmonary symptoms during acute infection [9,10].

Clinical Implications and Outcomes - Hypercoagulability in COVID-19

COVID-19 is characterized by a distinct hypercoagulable state with elevated D-dimer levels, fibrinogen abnormalities, and impaired fibrinolysis [9,10]. These abnormalities are attributed to endothelial injury and cytokine-mediated activation of coagulation pathways [9,10,14]. Elevated D-dimer levels have consistently been associated with severe disease and increased mortality [3,12].

Venous Thromboembolism and Pulmonary Embolism

Venous thromboembolism, including deep vein thrombosis and pulmonary embolism, is frequently reported in severe COVID-19 [16-18]. Pulmonary embolism can contribute to acute right ventricular failure and clinical deterioration [16-18].

Arterial Thrombosis and Acute Coronary Syndromes

Systemic inflammation and endothelial dysfunction can destabilize plaques and promote coronary thrombosis [20,21]. Arterial thrombosis may also occur via in situ thrombosis in a hypercoagulable state [9,10]. Differentiating type 1 myocardial infarction (MI) from type 2 MI and myocarditis is essential in patients with elevated biomarkers [19,28,29].

Microvascular Ischemia and Myocardial Injury

Microvascular thrombosis and endothelial inflammation contribute to myocardial injury independent of obstructive epicardial coronary disease [14]. Persistent microvascular dysfunction may contribute to prolonged myocardial impairment and post-acute risk [30].

Right Ventricular Dysfunction and Ischemic Stress

Right ventricular dysfunction is observed in severe disease, particularly with pulmonary embolism and pulmonary vascular involvement [16-18]. Echocardiographic studies report right ventricular dilation/dysfunction and associations with adverse outcomes [31]. COVID-19 has also been associated with the development of new cardiovascular pathology in previously healthy individuals [6,7,35,36]. Autopsy data further support the burden of thromboembolic disease in fatal cases [37].

Diagnostic evaluation of cardiovascular complications in COVID-19

Cardiac Biomarkers

The evaluation of cardiovascular complications in COVID-19 requires an integrated approach due to overlapping respiratory and cardiac manifestations [4,6]. Cardiac troponins are widely used for detecting myocardial injury, and elevations correlate with severity and mortality [3,12,22,24]. Natriuretic peptides may reflect ventricular dysfunction, right heart strain, or systemic inflammatory stress [11,12].

Electrocardiography

Electrocardiography remains essential for rhythm assessment and ischemia/pericarditis patterns, particularly in conjunction with biomarkers and imaging [20,21,23]. Electrocardiography (ECG) plays an important role in the evaluation of cardiovascular complications associated with COVID-19. Various abnormalities have been reported in affected patients, including ST-segment elevation or depression, T-wave inversion, conduction disturbances, and arrhythmias. These findings may reflect underlying myocardial injury, myocarditis, ischemia, or systemic inflammatory effects associated with SARS-CoV-2 infection.

Transthoracic Echocardiography

Transthoracic echocardiography evaluates ventricular function, wall motion abnormalities, and right ventricular strain. It is particularly useful in suspected myocarditis, new-onset heart failure, or pulmonary embolism [31,32].

Cardiac Magnetic Resonance Imaging

CMR is a key noninvasive tool for characterizing myocarditis and myocardial injury, using edema-sensitive imaging and late gadolinium enhancement [26,27,33]. ESC myocarditis statements and proposed diagnostic approaches provide clinical guidance [28,29].

Computed Tomography and Coronary Imaging

CT pulmonary angiography is the preferred modality for suspected pulmonary embolism [9,10]. Coronary imaging supports evaluation of acute coronary syndromes when invasive angiography is not immediately feasible [20,21].

Management strategies for COVID-19-associated cardiovascular complications

Management remains largely supportive and guided by standard cardiovascular treatment principles [4,6,9,10]. Continuous monitoring and optimization of oxygenation, fluid balance, and hemodynamics are essential, particularly in severe disease [4,6,11]. Patients with worsening or new-onset heart failure should generally receive guideline-directed medical therapy, continuing ACE inhibitors/angiotensin II receptor blockers (ARBs) and beta-blockers unless contraindicated [4,11,12].

Management is primarily supportive; severe cases may require ICU-level care. Clinical guidance and diagnostic/management frameworks are discussed in myocarditis statements and reviews [15,28,29,34]. Hospitalized patients should receive at least prophylactic anticoagulation when not contraindicated, with therapeutic anticoagulation for confirmed or strongly suspected thromboembolism [9,10]. ICU cohorts demonstrate high thrombotic risk despite prophylaxis [16,17]. Arrhythmias should be managed per standard guidelines with attention to hypoxia and electrolytes; atrial fibrillation is common in acute illness [23].

Short-term outcomes and prognosis

Several studies have demonstrated that cardiovascular involvement is associated with significantly worse clinical outcomes in patients with COVID-19. Myocardial injury, typically identified by elevated cardiac troponin levels, has been reported in approximately 20-30% of hospitalized patients and is associated with a two- to four-fold increase in mortality risk. Thromboembolic complications, including pulmonary embolism and deep vein thrombosis, have been observed in approximately 8-30% of patients with severe disease, particularly among those requiring intensive care. These findings highlight the important prognostic role of cardiovascular complications in COVID-19 and underscore the need for early recognition and appropriate management in hospitalized patients. Myocardial injury, elevated troponin levels, right ventricular dysfunction, and thromboembolic events are associated with increased ICU admission and mortality [3,9,12,16-18,31]. Patients with pre-existing cardiovascular disease are at particularly high risk [4-6,11,12]. CMR studies suggest that some patients demonstrate persistent myocardial abnormalities after recovery [26,27]. Long-term cohort data indicate increased post-acute cardiovascular risk following COVID-19 [30].

Limitations of current studies and future directions

This review has several limitations that should be acknowledged. First, the literature included in this narrative review primarily reflects studies published between 2020 and 2022, and therefore may not fully capture the most recent developments in the rapidly evolving field of COVID-19-related cardiovascular research. Second, as a narrative review, this study did not employ a formal systematic search strategy or quantitative meta-analytic methods, which may introduce selection bias in the studies discussed. Third, the available literature largely comprises retrospective observational studies conducted in the early phases of the pandemic, when diagnostic criteria, treatment protocols, and testing availability varied considerably across institutions. Finally, heterogeneity in the definitions of cardiovascular complications, particularly myocardial injury and myocarditis, may limit direct comparison between studies and affect the interpretation of reported outcomes [6,35,36]. Prospective studies are needed to clarify natural history, standardize definitions of cardiac involvement, and optimize anticoagulation strategies and long-term follow-up [9,10,30].

## Conclusions

COVID-19 is increasingly recognized as a systemic disease with important cardiovascular consequences. Evidence from the current literature demonstrates that myocardial injury, arrhythmias, heart failure, and thromboembolic complications represent major contributors to morbidity and mortality among hospitalized patients with SARS-CoV-2 infection. Emerging data suggest that these manifestations are driven by interconnected mechanisms, including endothelial dysfunction, thromboinflammation, immune-mediated myocardial injury, and microvascular damage. These processes contribute not only to acute cardiac complications but may also play a role in persistent cardiovascular abnormalities observed after recovery from the initial infection.

Early identification of cardiovascular involvement remains critical for improving outcomes. Integrated cardiovascular assessment, including cardiac biomarker evaluation, electrocardiography, and targeted imaging, may assist clinicians in identifying high-risk patients and guiding appropriate management strategies. Further research is needed to better define the long-term cardiovascular sequelae of COVID-19, optimize antithrombotic strategies, and clarify the natural history of post-infectious myocardial dysfunction. Improved understanding of these mechanisms will be essential for developing targeted therapeutic approaches and refining long-term cardiovascular care for patients affected by SARS-CoV-2 infection.

## References

[1] Singh TK, Zidar DA, McCrae K, Highland KB, Englund K, Cameron SJ, Chung MK (2023). A post-pandemic enigma: the cardiovascular impact of post-acute sequelae of SARS-CoV-2. Circ Res.

[2] Clerkin KJ, Fried JA, Raikhelkar J (2020). COVID-19 and cardiovascular disease. Circulation.

[3] Guo T, Fan Y, Chen M (2020). Cardiovascular implications of fatal outcomes of patients with coronavirus disease 2019 (COVID-19). JAMA Cardiol.

[4] Driggin E, Madhavan MV, Bikdeli B (2020). Cardiovascular considerations for patients, health care workers, and health systems during the COVID-19 pandemic. J Am Coll Cardiol.

[5] Nishiga M, Wang DW, Han Y, Lewis DB, Wu JC (2020). COVID-19 and cardiovascular disease: from basic mechanisms to clinical perspectives. Nat Rev Cardiol.

[6] Guzik TJ, Mohiddin SA, Dimarco A (2020). COVID-19 and the cardiovascular system: implications for risk assessment, diagnosis, and treatment options. Cardiovasc Res.

[7] Madjid M, Safavi-Naeini P, Solomon SD, Vardeny O (2020). Potential effects of coronaviruses on the cardiovascular system: a review. JAMA Cardiol.

[8] Zheng YY, Ma YT, Zhang JY, Xie X (2020). COVID-19 and the cardiovascular system. Nat Rev Cardiol.

[9] Bikdeli B, Madhavan MV, Jimenez D (2020). COVID-19 and thrombotic or thromboembolic disease: implications for prevention, antithrombotic therapy, and follow-up: JACC state-of-the-art review. J Am Coll Cardiol.

[10] Bader F, Manla Y, Atallah B, Starling RC (2021). Heart failure and COVID-19. Heart Fail Rev.

[11] Lala A, Johnson KW, Januzzi JL (2020). Prevalence and impact of myocardial injury in patients hospitalized with COVID-19 infection. J Am Coll Cardiol.

[12] Fox SE, Akmatbekov A, Harbert JL, Li G, Brown JQ, Heide RS (2020). Pulmonary and cardiac pathology in African American patients with COVID-19: an autopsy series from New Orleans. Lancet Respir Med.

[13] Ackermann M, Verleden SE, Kuehnel M (2020). Pulmonary vascular endothelialitis, thrombosis, and angiogenesis in COVID-19. N Engl J Med.

[14] Castiello T, Georgiopoulos G, Finocchiaro G (2022). COVID-19 and myocarditis: a systematic review and overview of current challenges. Heart Fail Rev.

[15] Helms J, Tacquard C, Severac F (2020). High risk of thrombosis in patients with severe SARS-CoV-2 infection: a multicenter prospective cohort study. Intensive Care Med.

[16] Klok FA, Kruip MJ, van der Meer NJ (2020). Incidence of thrombotic complications in critically ill ICU patients with COVID-19. Thromb Res.

[17] Poissy J, Goutay J, Caplan M (2020). Pulmonary embolism in patients with COVID-19: awareness of an increased prevalence. Circulation.

[18] Thygesen K, Alpert JS, Jaffe AS, Chaitman BR, Bax JJ, Morrow DA, White HD (2018). Fourth universal definition of myocardial infarction (2018). Circulation.

[19] Bangalore S, Sharma A, Slotwiner A (2020). ST-segment elevation in patients with COVID-19 - a case series. N Engl J Med.

[20] Stefanini GG, Montorfano M, Trabattoni D (2020). ST-elevation myocardial infarction in patients with COVID-19: clinical and angiographic outcomes. Circulation.

[21] Bonow RO, Fonarow GC, O'Gara PT, Yancy CW (2020). Association of coronavirus disease 2019 (COVID-19) with myocardial injury and mortality. JAMA Cardiol.

[22] Saha SA, Russo AM, Chung MK, Deering TF, Lakkireddy D, Gopinathannair R (2022). COVID-19 and cardiac arrhythmias: a contemporary review. Curr Treat Options Cardiovasc Med.

[23] Lippi G, Lavie CJ, Sanchis-Gomar F (2020). Cardiac troponin I in patients with coronavirus disease 2019 (COVID-19): evidence from a meta-analysis. Prog Cardiovasc Dis.

[24] Boehmer TK, Kompaniyets L, Lavery AM (2021). Association between COVID-19 and myocarditis using hospital-based administrative data - United States, March 2020-January 2021. MMWR Morb Mortal Wkly Rep.

[25] Puntmann VO, Carerj ML, Wieters I (2020). Outcomes of cardiovascular magnetic resonance imaging in patients recently recovered from coronavirus disease 2019 (COVID-19). JAMA Cardiol.

[26] Kotecha T, Knight DS, Razvi Y (2021). Patterns of myocardial injury in recovered troponin-positive COVID-19 patients assessed by cardiovascular magnetic resonance. Eur Heart J.

[27] Caforio AL, Pankuweit S, Arbustini E (2013). Current state of knowledge on aetiology, diagnosis, management, and therapy of myocarditis: a position statement of the European Society of Cardiology Working Group on Myocardial and Pericardial Diseases. Eur Heart J.

[28] Siripanthong B, Nazarian S, Muser D (2020). Recognizing COVID-19-related myocarditis: the possible pathophysiology and proposed guideline for diagnosis and management. Heart Rhythm.

[29] Xie Y, Xu E, Bowe B, Al-Aly Z (2022). Long-term cardiovascular outcomes of COVID-19. Nat Med.

[30] Spetz M, Dag YN, Li H, Nyberg F, Rosvall M (2025). COVID-19 and cardiovascular disease in a total population-study of long-term effects, social factors and COVID-19-vaccination. Nat Commun.

[31] Dweck MR, Bularga A, Hahn RT (2020). Global evaluation of echocardiography in patients with COVID-19. Eur Heart J Cardiovasc Imaging.

[32] Skulstad H, Cosyns B, Popescu BA (2020). COVID-19 pandemic and cardiac imaging: EACVI recommendations on precautions, indications, prioritization, and protection for patients and healthcare personnel. Eur Heart J Cardiovasc Imaging.

[33] Friedrich MG, Sechtem U, Schulz-Menger J (2009). Cardiovascular magnetic resonance in myocarditis: a JACC white paper. J Am Coll Cardiol.

[34] Lee CC, Ali K, Connell D, Mordi IR, George J, Lang EM, Lang CC (2021). COVID-19-associated cardiovascular complications. Diseases.

[35] Long B, Brady WJ, Koyfman A, Gottlieb M (2020). Cardiovascular complications in COVID-19. Am J Emerg Med.

[36] Lee S, Hwang SH, Park S (2025). Burden of cardiovascular outcomes after SARS-CoV-2 infection in South Korea and Japan: a binational population-based cohort study. Circulation.

[37] Wichmann D, Sperhake JP, Lütgehetmann M (2020). Autopsy findings and venous thromboembolism in patients with COVID-19: a prospective cohort study. Ann Intern Med.

